# Exploring the Nutritional Potential of Spent Coffee Grounds as a Substitute for Rice Bran in Feeds for Nile tilapia, *Oreochromis niloticus*: An Evaluation of Growth Performance and Biological Indices

**DOI:** 10.1155/2024/4858465

**Published:** 2024-03-30

**Authors:** Wikit Phinrub, Sontaya Sookying, Phanit Srisuttha, Nantaporn Sutthi, Paiboon Panase

**Affiliations:** ^1^Department of Aquaculture and Fishery Products Faculty of Science and Fisheries Technology, Rajamangala University of Technology Srivijaya, Trang Campus, Trang 92150, Thailand; ^2^Unit of Excellence “Physiology and Sustainable Production of Terrestrial and Aquatic Animals” Division of Fisheries, School of Agriculture and Natural Resources, University of Phayao, Phayao 56000, Thailand; ^3^Division of Pharmacy and Technology, Department of Pharmaceutical Care, School of Pharmaceutical Sciences, University of Phayao, Phayao 56000, Thailand; ^4^Division of Applied Thai Traditional Medicine, School of Public Health, University of Phayao, Phayao 56000, Thailand; ^5^Department of Agricultural Technology, Faculty of Technology, Mahasarakham University, Mahasarakham 44150, Thailand; ^6^Division of Fisheries, School of Agriculture and Natural Resources, University of Phayao, Phayao 56000, Thailand

## Abstract

This study aimed to assess the viability of replacing rice bran with spent coffee grounds (SCG) in the diets of *Oreochromis niloticus* (average body weight, 48.8 ± 0.42 g). The fish were randomly allocated into four sets of three groups each and placed in net cages (1 m × 2 m × 0.5 m) at a density of 30 fish per cage. They were fed diets with four different replacement levels: 0%, 5%, 10%, and 15% of SCG over a period of 90 days. Growth and serum biochemical indices were monitored three times at 30, 60, and 90 days. During the experiment, there were no significant differences (*P*  > 0.05) observed in growth indices, including weight gain (WG), daily WG, specific growth rate, feed conversion rate, protein efficiency ratio, and survival rate among the groups at 30, 60, and 90 days. Serum biochemical indices, such as aspartate aminotransferase and alanine aminotransferase, showed a similar trend with significant differences observed only on day 30, while the lowest and highest levels were found in the control and 15% SCG replacement groups, respectively. For total cholesterol, a significantly different result was found only on day 30. However, these differences were not sustained in subsequent assessments. Conversely, serum glucose, total protein, albumin, and globulin remained unaffected by SCG replacement throughout the experiment. The findings indicate that replacing rice bran with up to 15% SCG did not adversely impact the growth performance or key serum biochemical indices of Nile tilapia. To the researchers' knowledge, these findings are the first in the field to substitute SCG for rice bran, opening a new avenue for further research.

## 1. Introduction

The contemporary world is marked by rapid economic and technological progress, resulting in increased generation of both mineral and organic waste, thus necessitating environmentally responsible management as an integral component of the circular economy [1]. Coffee is one of the most in-demand global commodities, with widespread consumption on a worldwide scale, as evidenced by the consumption of approximately 10.2 million tons of coffee between 2020 and 2021 [2]. Spent coffee grounds (SCG) is the residue produced during the brewing process and is the primary byproduct resulting from the preparation of coffee beverages; for each kilogram of green coffee, about 0.65 kg of SCG are generated, and roughly 2 kg of wet SCG are produced for every kilogram of instant coffee. Unfortunately, most of this byproduct is incinerated or sent to landfills [3]. SCG serves as a prime illustration of organic waste necessitating efficient environmental management to avoid environmental pollution. Additionally, SCG may contain antinutritional factors such as starch polysaccharides and nonstarch polysaccharides, some of which could hinder their suitability for animal feed [4]. Nonetheless, SCG has the potential to be a viable alternative for animal feed due to its significant organic content and valuable nutrient composition, while it also serves as a source xof neutral detergent fiber, protein, and lipids, while it also notably contains a significant quantity of polyunsaturated fatty acids (PUFAs) [5, 6]. Nevertheless, Campos-Vega et al. [7] showed that SCG, along with other coffee byproducts, contains valuable bioactive compounds that can potentially be extracted and repurposed, such as polyphenols, accounting for approximately 1%–1.5% of total polyphenols. Consequently, xnumerous recent studies have shifted toward exploring alternative applications for SCG. The literature review revealed a conspicuous dearth of information regarding the effects of SCG on aquatic animal production. The findings in this study are based primarily on the report by Van Doan et al. [8], which indicated that adding 10%–80% SCG to tilapia diets reared in a biofloc system resulted in ideal growth and immune responses when using a 10% supplementation. However, the serum biochemical indices in experimental fish were not reported. Still, it is worth noting that previous research concerning the potential of SCG as a substitute for specific feed ingredients, particularly rice bran, is conspicuously lacking.

Rice bran is a frequently utilized ingredient in animal feed formulations, including those for fish. Rice bran boasts an average protein content of around 13.35% [9], similar to coffee grounds, which typically has protein values between approximately 10% and 17% [10]. Rice bran typically costs around 35–45 Thai baht per kilogram (equivalent to 1.0–1.5 USD per kilogram). In contrast, SCG are readily available at no cost since they can be obtained from coffee roasting factories in Phayao Province, Thailand.

*Oreochromis niloticus* plays a crucial role in enhancing the livelihoods of communities, particularly in developing nations, through its widespread cultivation as one of the most commonly farmed fish worldwide. The annual production of farmed tilapia exceeds 6 million tonnes, solidifying its position as the second most extensively cultivated freshwater fish globally, trailing only behind carps [11]. Meanwhile, FAO [12] has documented that Nile tilapia ranks as the third most cultivated freshwater fish worldwide, following Grass carp (*Ctenopharyngodon idellus*) and Silver carp (*Hypophthalmichthys molitrix*), respectively. In 2022, Nile tilapia was the top freshwater fish culture in Thailand, accounting for 57.7% of all fish species, with a total quantity of 269,400 tonnes valued at 12,613.9 million Thai baht (354.3 million USD) [13]. Based on the economic importance of this fish species, reducing the cost of feed is considered a crucial factor for aquaculture in Thailand.

Hence, this research aims to assess the feasibility of incorporating SCG as a dietary substitution for rice bran in the feed of Nile tilapia (*O. niloticus*). The study focuses on monitoring both the growth performance and serum biochemistry indices in Nile tilapia over 3 months. In particular, serum biochemical parameters were investigated every month throughout the feeding trials.

## 2. Materials and Methods

### 2.1. Preparation of SCG

SCG were sourced from coffee roasting facilities in Phayao Province, Thailand. To prepare the SCG for analysis, the procedures adjusted by Van Doan et al. [8] involved soaking them in water for 25 hr, followed by drying at 60°C in an incubator for 72 hr. The SCG were subsequently pulverized into a fine powder and underwent proximate analysis, which involved assessing the levels of moisture, ash, crude protein, fiber, and crude fat. The examination adhered to the AOAC [14] standard protocols. Moisture content was evaluated using the Kjeldahl distillation method, and crude lipid (fat) content was established via Soxhlet extraction. The total ash content was quantified using appropriate drying methods, and fiber content was analyzed using the Gerhardt Fibre Bag System (Table 1). After analysis, the SCG powder was stored in sterile plastic containers at room temperature for future research purposes, with a stipulation that it should not be stored for longer than 1 month to maintain its quality and integrity.

### 2.2. Fish-Feed Formulations and Experimental Design

Commercial fish stuffs were acquired from an agricultural supply store located in Chiang Mai, Thailand. Four distinct levels of rice bran replacement by SCG powder were created at 0% (control group), 5%, 10%, and 15%, respectively, using proximate analysis following the methods outlined above (Table 2). For the experimental phase, juvenile Nile tilapia with an average body weight of 48.8 ± 0.42 g were procured from a commercial fish farm (Chok-Anan farm) in Chiang Rai, Thailand. A 2-week acclimatization period in a controlled laboratory environment within a cement pond (3 m × 5 m × 0.8 m) was conducted. Throughout this period, water quality was regularly assessed at 7:00 am. Water temperature was upheld at 29.3 ± 1.32°C, pH at 7.3 ± 0.31, dissolved oxygen concentration at 7.3 ± 0.27 mg/l, and all measured using multiprobes (HORIBA, U50 series, Japan) according to APHA guidelines [15]. To aid in the acclimatization process within the cement ponds, water exchanges were conducted every 7 days, with 50% of the total water volume being replaced each time. A completely randomized design experiment was utilized. A sum of 480 robust fish was divided into four sets of three replicates, with each set containing 30 fish per replicate. These groups were reared in 12 net cages (1 m × 2 m × 0.5 m), all positioned within the same earthlike pond. Each group was provided with its designated diet as previously described; at the start of the experiment in the first month, we supplied a daily quantity equivalent to 7% of their body weight, which was subsequently reduced to 5% and 3% in the second and third months, respectively.

### 2.3. Growth Performance, Feed Utilization, and Survival

Fish in every experimental group were weighed at 15-day intervals to allow for modifications in the amount of feed provided. Following that, growth metrics were computed, and the findings were recorded in reports issued every 30 days, specifically at the 30, 60, and 90 days. The growth parameters, such as weight gain (WG), average daily gain (ADG), specific growth rate (SGR), feed conversion rate (FCR), survival rate (SR), and protein efficiency ratio (PER), were calculated following the formulas provided by Abdel Raman et al. [16]:

(1) $$\begin{array}{c} \text{WG}=\text{ Final weight }\left(\text{g}\right)-\text{ initial weight }\left(\text{g}\right), \end{array}$$

(2) $$\begin{array}{c} \text{ADG}=\frac{\left[\text{Final weight }\left(\text{g}\right)-\text{ initial weight }\left(\text{g}\right)\right]}{\text{ Experimental days}}, \end{array}$$

(3) $$\begin{array}{c} \text{SGR}=\frac{\left\{\text{Ln final weight }\left(\text{g}\right)-\text{ Ln initial weight }\left(\text{g}\right)\right\}}{\text{ Experimental days}}\times 100, \end{array}$$

(4) $$\begin{array}{c} \text{FCR}=\frac{\text{ Total feed fed }\left(\text{g}\right)}{\text{ Weight gain }\left(\text{g}\right)}, \end{array}$$

(5) $$\begin{array}{c} \text{SR}=\frac{\left[\text{Number of surviving fish}\right]}{\text{ Initial number of fish}}\times 100, \end{array}$$

(6) $$\begin{array}{c} \text{PER}=\frac{\text{ Wet weight gain }\left(\text{g}\right)}{\text{Crude protein fed}\left(\text{g}\right)}. \end{array}$$

### 2.4. Sampling and Serum Biochemistry Assays

At 30-day intervals at 30, 60, and 90 days throughout the research period, 36 fish were anesthetized using MS-222 in dechlorinated water at 1 : 4,000 v/v [17], and three fish were selected randomly from each cage/replication. Carefully, blood samples, totaling 1.0 ml each, were gathered from the caudal blood vessels of each fish utilizing syringes devoid of heparin blood samples were left at room temperature for 4 hr to facilitate clot formation. After collection, the serum was separated by centrifuging the samples at 5,000 rpm for 15 min. Once separated, the serum samples were preserved at 20°C until they were ready for analysis, with a maximum storage period not exceeding 7 days. Thereafter, serum samples were subsequently sent to the Chiang Mai Veterinary Laboratory Centre Limited Partnership in Chiang Mai, Thailand, for analysis. A wide array of biochemical markers were examined, encompassing alanine aminotransferase (ALT), aspartate aminotransferase (AST), alkaline phosphatase (ALP), creatinine, glucose, albumin, globulin, total protein, triglycerides, and cholesterol. These assessments were performed utilizing a medical chemistry analyzer, the Pentra C400 model, manufactured by HORIBA, Japan [18].

### 2.5. Statistical Analysis

Before examining the growth and serum indices, the data were normalized and checked for homogeneity of variance. Then, all parameters underwent a one-way analysis of variance, followed by Tukey's post hoc test, with a confidence level of 95% (*P*  < 0.05). The findings are displayed as the mean ± standard deviation (SD). Statistical analyses were executed using SPSS software version 17 for Windows, developed by SPSS Inc., Chicago, USA.

## 3. Results

### 3.1. Growth and SR

**Figure 1:**
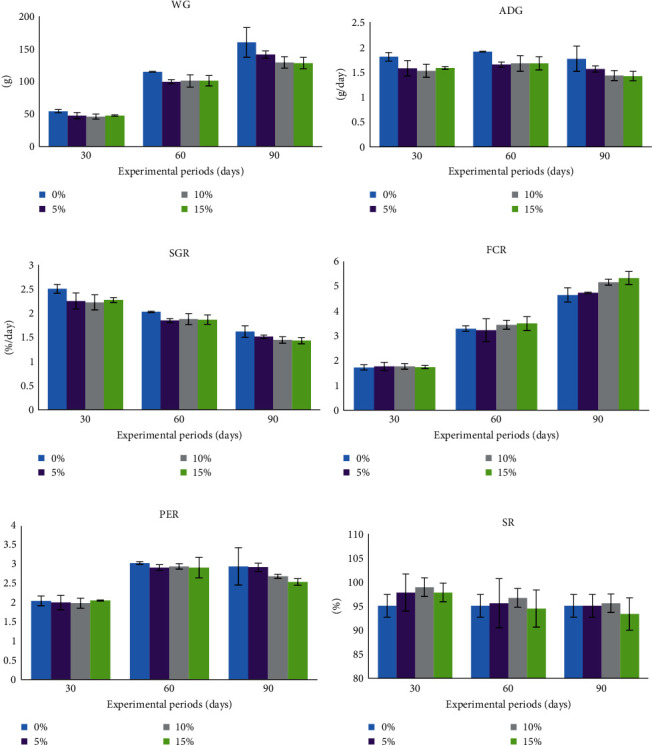
Effects of rice bran replacement by different levels of spent coffee grounds (SCG) on growth performance of Nile tilapia (*O. niloticus*) reared in net cages for 90 days. WG = weight gain (a), ADG = average daily growth (b), SGR = specific growth rate (c), FCR = feed conversion rate (d), PER = protein efficiency ratio (e), and SR = survival rate (f).

During the 90-day experimental period, there were no significant differences (*P* > 0.05) observed in the growth parameters of *O. niloticus* compared to the control group on days 30, 60, and 90. On the 60 days of the experiment, variations in WG, ADG, and SGR were initially noted, with all experimental groups exhibiting a declining pattern compared to the control group. This trend continued on day 90, with the treatment groups exhibiting a decreasing trend compared to the control group. However, no statistically significant differences were found. Divergences in FCR were noted at days 60 and 90 of the experiment, with the groups that received 10% and 15% rice bran replacement by SCG displaying a rising trend in comparison to both the control group and the group that received 5% rice bran replacement by SCG. Nonetheless, no statistically significant variances were observed (*P* > 0.05). Additionally, there were no statistically significant differences in SR on days 30, 60, and 90 of the experiment. However, the group receiving 15% rice bran replacement by SCG had the lowest value compared to the other groups (Figure 1(a)–1(f)).

### 3.2. Biochemical Indices

**Figure 2:**
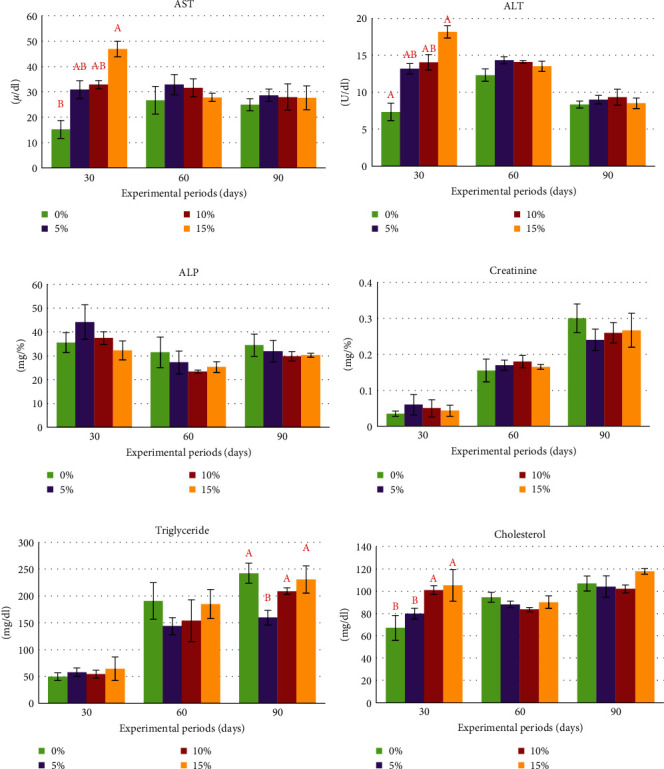
Effects of rice bran replacement by different levels of spent coffee grounds (SCG) on serum biochemistry indices of Nile tilapia (*O. niloticus*) reared in net cages for 90 days. AST; aspartate transaminase (a), ALT; alanine transaminase (b), ALP; alkaline phosphatase (c), creatinine (d), triglyceride (e), and total cholesterol (f).

**Figure 3:**
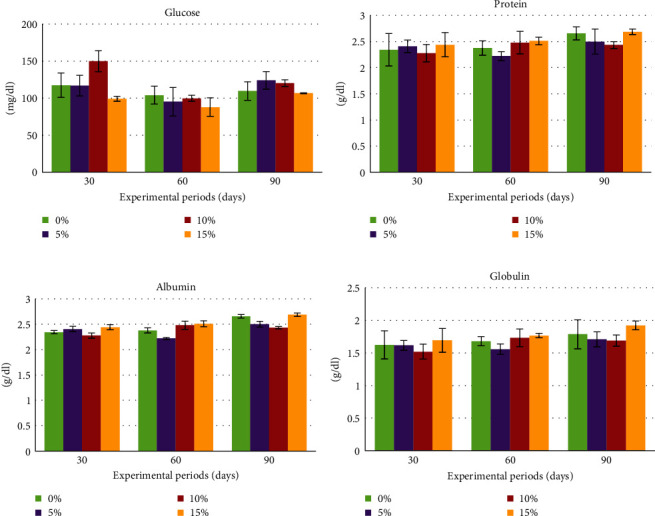
Effects of rice bran replacement by different levels of spent coffee grounds (SCG) on serum biochemistry indices of Nile tilapia (*O. niloticus*) reared in net cages for 90 days; glucose (a), total protein (b), albumin (c), and globulin (d).

During the experiment, serum biochemical indices, including AST, ALT, ALP, creatinine, triglyceride, glucose, total protein, albumin, and globulin, were assessed on days 30, 60, and 90. Notably, AST and ALT exhibited a similar pattern, with a significant difference (*P*  < 0.05) observed only on day 30. At this time, the control group displayed the lowest levels of AST and ALT, while the other groups showed a significant increase in these enzymes with higher percentages of rice bran replacement. Nonetheless, these indices did not exhibit statistically significant differences after day 30 (Figures 2(a) and 2(b)). Meanwhile, levels of serum ALP, creatinine, and triglycerides did not exhibit significant differences compared to those of the control group. It is noteworthy that on days 60 and 90 of the experiment, all treatment groups had lower values than the control group. Cholesterol showed statistically significant differences only during the initial 30 days of the experiment, but thereafter, no statistical differences were observed from day 60 to day 90 of the experiment (Figure 2(c)–2(f)). Glucose levels appeared to differ on day 30 of the experiment, with higher levels observed in the group receiving 10% rice bran replacement by SCG compared to the other groups, but no statistically significant differences were found. However, on days 60 and 90, all groups showed similar values. Meanwhile, total protein, albumin, and globulin did not exhibit statistically significant differences throughout the study (Figure 3(a)–3(d)).

## 4. Discussion

The aim of this study was to assess the nutritional value of Nile tilapia feed by substituting rice bran with SCG, thereby exploring a sustainable approach to feed formulation in aquaculture. There are two key findings in our study. First, SCG can be used as a substitute for rice bran as demonstrated by no significant changes in growth performance. Second, the serum biochemical indices suggest that up to 15% of rice bran can be safely and effectively replaced by SCG. Based on the proximate analysis results, it was observed that SCG exhibited protein levels comparable to those of rice bran (13.35% protein) [9]. Our findings provide preliminary evidence that SCG could potentially be a viable candidate for further evaluation as a potential raw material for formulating aquatic animal feed, given that SCG is a valuable resource containing significant amounts of chlorogenic acid, caffeine, bioactive peptides, phenolic acids, and flavonoids [19–22].

As such, the utilization of SCG byproducts is a promising and useful candidate to promote a circular economy. This study involved replacing rice bran with various percentages of SCG ranging from 0% to 15%. The findings of this study indicate that there were no observed adverse effects on the growth indices of *O. niloticus* over the 90-day experimental period. To date, there have been limited reports on the application of SCG in aquatic animal feed, with the only documented study on this subject being conducted by Van Doan et al. [8], which reported that incorporating 10% SCG into the diet of *O. niloticus* reared in a biofloc system has the potential to serve as an immunostimulant in tilapia aquaculture. However, the previous study resulted in a decline in growth performance when higher levels of SCG were included in their diets. This may be due to elevated levels of SCG in the diet, resulting in a notable decrease in growth rate and an increase in the feed conversion ratio. This effect may be attributed to nonenzymatic reactions between amino acids and reducing sugars that occur when coffee beans are processed, ultimately leading to reduced nutrient digestion [23]. According to reports on livestock, SCG fermented by *Lactobacillus plantrum* can improve growth performance more than non-fermented SCG because nitrogen quality can be improved, as seen in Choi et al. [6]. Additionally, SCG had a beneficial impact on blood antioxidant status, characterized by an enhancement in ferric-reducing antioxidant power and a reduction in malondialdehyde levels. However, the supplementation of SCG did not influence the antioxidant status of milk in dairy goats [24]. In the present study, it was observed that the subjects' growth performance did not show a positive impact when rice bran was replaced by SCG, but importantly, no adverse effects were observed. A potential explanation for this could be that a lower volume of SCG was added to the diet formulations compared to previous studies. The constraint on increasing the amount of SCG in aquatic animal diets could be attributed to the Maillard reaction. This process, which occurs during bean roasting, involves a non-enzymatic chemical interaction between amino acids and reducing sugars. Consequently, the nutrient digestibility of the beans is reduced [25]. Improving the nutritional value of SCG for animal feed can be achieved through fermentation using microorganisms such as *Lactobacillus plantarum*, as demonstrated by Choi et al. [6]. Microbes are able to degrade lignin or disrupt the bonds between lignin and cellulose within the cell walls of plants [25]. As indicated by Sarghini et al. [26], another viable approach involves subjecting SCG to acid hydrolysis. These methods have shown efficacy in enhancing the benefits of SCG as animal feed ingredients. This enhancement results in improved animal growth performance. Based on our research findings, it is observed that SCG can be utilized as a viable alternative to rice bran, with a potential replacement rate of up to 15%, which resulted in no negative impact on overall growth performance. To further validate these results, a comprehensive serum biochemistry analysis was conducted over a period of 90 days during the experiment.

Assessing fish health status often relies on examining biochemical parameters in their blood [27, 28]. Serum biochemical indices were evaluated, including ALT, AST, ALP, creatinine, glucose, albumin, globulin, total protein, triglycerides, and cholesterol. The thorough literature review found no previous studies that investigated the impact of SCG on serum biochemical parameters in aquatic animals, specifically in Nile tilapia. Consequently, this study sought to fill this research gap by exploring the effects of SCG on the aforementioned serum parameters in Nile tilapia that are reared in net cages. When the liver is stressed or damaged, liver impairment or tissues undergo damage or dysfunction [29]. AST and ALT are crucial enzymes involved in the release of substances into the bloodstream, which significantly affect the metabolism of proteins and amino acids [30, 31]. Throughout this study, both ALT and AST values consistently demonstrated similar patterns throughout the experiment. Interestingly, distinct variations were observed in the initial 30 days, which may be linked to the organisms adjusting to a novel nutritional status, resulting in physiological changes or an initial stress response known as the alarm phase [32]. However, no further disparities were identified at 60 and 90 days into the experiment, potentially indicating the restoration of normal body function attributed to the nontoxic nature of SCG as a feed ingredient. By contrast, rainbow trout (*Oncorhynchus mykiss*) exhibited a linear increase in both AST and ALT levels over time when subjected to sub-lethal concentrations of cadmium (1 and 3 *μ*g/l) for a duration of 30 days, correlating with the cadmium concentration [33]. Meanwhile, for American eel (*Anguilla rostrata*), the provision of compound acidifiers (ACs) supplementation in their diet resulted in a significant reduction in the activities of ALT and AST in the serum. This implies that the addition of dietary CAs could be advantageous in enhancing the liver health of juvenile *A. rostrata* [34]. ALP serves as a versatile enzyme, acting as a transphosphorylase under alkaline conditions. Its crucial involvement extends to the mineralization of the skeletal structures in aquatic animals and contributes significantly to membrane transport activities [35]. The current research results indicate that incorporating SCG into the diets did not negatively impact the health condition of *O. niloticus*. Nonetheless, the quantity of SCG incorporated into the diet may be insufficient to elicit a positive shift in ALP level, as documented by Zhang et al. [34], which revealed that the addition of 2–3 g/kg of compound acidifiers to the feed did not influence ALP. However, a statistically significant increase in ALP level was observed when the supplementation level was increased to 5 g/kg.

Triglycerides and cholesterol function as the primary energy storage components in fish, and they are synthesized in excess quantities during periods of stress [36]. The findings of this study indicate that the group fed a 5% replacement demonstrated a significantly lower level of triglyceride at day 90, which returned to normal when a higher percentage of replacements were introduced. This phenomenon may be attributed to the enriching PUFAs in SCG, known for their association with improved lipid profiles [37]. Mota et al. [38] presented findings on the fatty acid compositions in SCG, revealing that total saturated fatty acids, PUFAs, and monounsaturated fatty acids (MUFA) comprised 52.1%, 36.8%, and 11.1%, respectively. In a separate study, Iriondo-DeHond et al. [39] identified 45.19% MUFA in SCG. Additionally, Danh et al. [40] reported that SCG contained linoleic acid (omega-6) and *α*-linolenic acid (omega-3) at 44% and 1% of the total fatty acid composition, respectively. Supporting the results of this study, a meta-analysis has shown that n-3 PUFAs can effectively reduce serum triglyceride levels in individuals with metabolic syndrome [41]. The adequate intake of n-3 PUFAs has a preventative role in hypertriglyceridemia by inhibiting the production of triglycerides and low-density lipoproteins by the liver [42]. Moreover, under conditions of positive energy balance, n-3 PUFAs support the growth of fat cells and the expansion of adipose tissue, ultimately fostering a favorable metabolic profile [41]. While the mechanism behind the reduction of triglycerides by omega-3 fatty acids has not been fully elucidated, Rundblad et al. [43] shed light on the details of this action. Nevertheless, a significant difference in total cholesterol levels was only found during the initial investigation on day 30. No significant differences were observed in the subsequent assessments. Liu et al. [41] showed that PUFAs did not significantly reduce total cholesterol or LDL-c levels in the serum of patients with metabolic syndrome.

The concentrations of total protein, albumin, and globulin in serum are indicative of the metabolic status, absorption of amino acids or peptides, and the nonspecific immune function of fish [44, 45]. Throughout the duration of the study, no noteworthy variations were detected. This suggests that the supplementation of SCG in Tilapia diets did not result in adverse effects, and the nonspecific immune function remained normally active. Notably, the addition of smaller amounts of SCG supplements in this study (maximum, 2.25% in the diet) did not result in positive effects for these parameters. In contrast, Van Doan et al. [8] demonstrated that a 10% supplementation had favorable effects on SGR and FCR, as well as notable improvements in skin mucosal and serum immunities in Nile tilapia. However, the addition of higher levels of SCG resulted in a decline of these parameters.

## 5. Conclusion

The findings of this study suggest that replacing rice bran with SCG could be viable up to 15% (2.25 g/100 g) of their diet without any detrimental impact on growth performance or the key parameters assessed in this study. While the main serum parameters were not influenced by substitution with SCG, these effects were not consistently significant throughout the entire duration of the experiment. Nonetheless, further studies with varying replacement percentages of SCG should be conducted to determine the optimal dosage for Nile tilapia culture and investigate potential uses for various commercially farmed aquatic species, with a specific emphasis on reducing feed costs.

## Figures and Tables

**Table 1:** Proximate composition (in % dry matter) of spent coffee ground meal.

Proximate compositions	Spent coffee grounds (% in dry weight)
Moisture	3.31
Ash	7.17
Protein	15.12
Lipid	11.96
Fiber	24.19

*Note*: Values are expressed as mean ± SD.

**Table 2:** Ingredients and proximate composition of the four experimental diets.

Ingredient compositions (%)	Experimental diets (dry weight)			
	0%	5%	10%	15%
Commercial fish meal	25.15	25.15	25.15	25.15
Soybean meal	25.15	25.15	25.15	25.15
Corn starch	14.9	14.9	14.9	14.9
Broken rice	14.9	14.9	14.9	14.9
Rice bran	14.9	14.15	13.41	12.67
Spent coffee grounds	0	0.77	1.49	2.25
Premix	5	5	5	5
Total (%)	100	100	100	100
*Proximate composition* (% in dry weight)				
Moisture	10.45	10.04	9.13	9.41
Ash	15.54	16.06	14.56	16.85
Protein	31.02	32.03	31.41	32.41
Lipid	8.59	8.23	8.87	8.33
Fiber	33.43	27.27	27.74	28.18

*Note*: Premix 1 kg contained *vitamins*, including vitamin A 36,000 IU, vitamin D3 9,000 IU, vitamin E 187 mg, vitamin K 19 mg, vitamin B1 52 mg, vitamin B2 97 mg, vitamin B6 46 mg, vitamin C (coated) 69,800 mg activity, vitamin B12 60 *µ*g, pantothenic acid 93 mg, niacin 130 mg, folic acid 10 mg, Inositol 225 mg, and biotin 450 *µ*g, as well as *minerals*, including Mg 105 mg, Cu 9 mg, Fe 90 mg, Zn 90 mg, I 1.8 mg, Co 450 *µ*g, Mg 1,900 mg, Se 150 *µ*g, Na 117 mg, K 3,600 mg, and Ca 219 mg.

## Data Availability

The data were not deposited in a public repository. Data are available upon reasonable request directly from the corresponding authors.
